# Multiple mutations across the succinate dehydrogenase gene complex are associated with boscalid resistance in *Didymella tanaceti* in pyrethrum

**DOI:** 10.1371/journal.pone.0218569

**Published:** 2019-06-20

**Authors:** Tamieka Lee Pearce, Calum Rae Wilson, David Hugh Gent, Jason Barry Scott

**Affiliations:** 1 Horticulture Centre, Tasmanian Institute of Agriculture, University of Tasmania, Burnie, Tasmania, Australia; 2 Horticulture Centre, Tasmanian Institute of Agriculture, University of Tasmania, Sandy Bay, Tasmania, Australia; 3 Forage Seed and Cereal Research Unit, United States Department of Agriculture—Agricultural Research Service, Corvallis, Oregon, United State of America; Universita degli Studi di Pisa, ITALY

## Abstract

Failures in control of tan spot of pyrethrum, caused by *Didymella tanaceti*, has been associated with decreased sensitivity within the pathogen population to the succinate dehydrogenase inhibitor (SDHI) fungicide boscalid. Sequencing the *Sdh*B, *Sdh*C, and *Sdh*D subunits of isolates with resistant and sensitive phenotypes identified 15 mutations, resulting in three amino acid substitutions in the SdhB (H277Y/R, I279V), six in the SdhC (S73P, G79R, H134R, H134Q, S135R and combined H134Q/S135R), and two in the SdhD (D112E, H122R). *In vitro* testing of their boscalid response and estimation of resistance factors (RF) identified isolates with wild-type (WT) Sdh genotypes were sensitive to boscalid. Isolates with SdhB-I279V, SdhC-H134Q and SdhD-D112E exhibited moderate resistance phenotypes (10 ≥ RF < 100) and isolates with SdhC-H134R exhibited very high resistance phenotypes (RF ≥ 1000). All other substitutions were associated with high resistance phenotypes (100 ≥ RF < 1000). High-resolution melt assays were designed and used to estimate the frequencies of substitutions in four field populations (*n* = 774) collected in August (pre-boscalid application) and November (post-boscalid application) 2012. The SdhB-H277Y, SdhC-H134R and SdhB-H277R genotypes were most frequently observed across populations at 56.7, 19.0, and 10.3%, respectively. In August 92.9% of *D*. *tanaceti* contained a substitution associated with decreased sensitivity. Following boscalid application, this increased to 98.9%, with no WT isolates detected in three fields. Overlaying previously obtained microsatellite and mating-type data revealed that all ten recurrent substitutions were associated with multiple genotypes. Thus, boscalid insensitivity in *D*. *tanaceti* appears widespread and not associated with clonal spread of a limited pool of individuals.

## Introduction

Foliar and flower disease control in pyrethrum (*Tanacetum cinerariifolium*) production involves the application of a range of fungicide chemistries with timing designed to optimise the production of flowers in early austral summer. Flowers are the sole harvested product of the crop, from which pyrethrins are extracted for their insecticidal properties [1, 2]. Historically, spring fungicide applications to control ray blight, caused by *Stagonosporopsis tanaceti*, have been the main focus for disease management [3–5]. However, surveys conducted during 2012 and 2013 indicated that *S*. *tanaceti* had been displaced as the predominant foliar pathogen of pyrethrum in spring by *Didymella tanaceti* [6, 7], the causal agent of tan spot and previously regarded as of minor concern for the industry [8]. The increased prevalence of *D*. *tanaceti* coincided with failures of the spring fungicide program to effectively control foliar diseases and the detection of resistance to the succinate dehydrogenase inhibitors (SDHI) fungicide boscalid within *D*. *tanaceti*, but not *S*. *tanaceti* [7]. Boscalid has been a key component of the spring fungicide program since 2005 [5, 9]. It is theorised that fungicide resistance has provided a competitive advantage to *D*. *tanaceti* over *S*. *tanaceti*.

The development of fungicide resistance in a pathogen population is a major concern for any disease management system. Fungicides with single site modes of action, such as the SDHI, are particularly prone to the development of resistance [10, 11]. Succinate dehydrogenase is an enzyme necessary for cellular respiration [12] and is constructed from four nuclear encoded protein subunits; SdhA, SdhB, SdhC and SdhD [10, 13, 14]. The SdhB subunit contains three iron-sulphur clusters (2Fe-2S, 4Fe-4S and 3Fe-4S) which transfer electrons for the reduction of ubiquinone. The ubiquinone binding site (Q-site) is formed by the interface of the SdhB, SdhC and SdhD subunits [12, 13, 15, 16]. Boscalid inhibits ubiquinone reduction by binding to the Q-site, restricting cellular respiration and resulting in cell death. Boscalid has been used for disease control in a wide range of agricultural cropping systems, such as *Alternaria alternata* [17, 18], *Botrytis cinerea* [19, 20], and *Sclerotinia sclerotiorum* [21, 22] in several crops. However, use of boscalid has been associated with increasingly frequent reports of resistance in pathogen populations. Mutations within each of the subunits have been correlated with boscalid resistance within field isolates and laboratory-induced mutants of different fungal species [10, 23]. Early reports of such instances include *A*. *alternata* in pistachio [18] and *Didymella bryoniae* in watermelon [24]. Resistance development has also been recorded despite application strategies to minimise this risk, such as tank mixing with multi-site protectants and limiting application numbers [25].

The purpose of this study was to examine the genetic basis for boscalid resistance in *D*. *tanaceti*. It was hypothesised that boscalid resistance would be associated with mutations within the succinate dehydrogenase (*Sdh*) gene complex. To this end, sequencing of the *Sdh*B, *Sdh*C and *Sdh*D genes was conducted for *D*. *tanaceti* individuals with known boscalid response phenotypes. A secondary objective of this study was to examine the influence of a single application of boscalid in commercial pyrethrum production on the abundance and genotype of boscalid-resistant pathogen isolates. To facilitate this, a high-resolution melt (HRM) assay was developed for the detection of known and potential unknown mutations associated with boscalid resistance.

## Results

### *Sdh*B, *Sdh*C and *Sdh*D sequences

**Fig 1:**
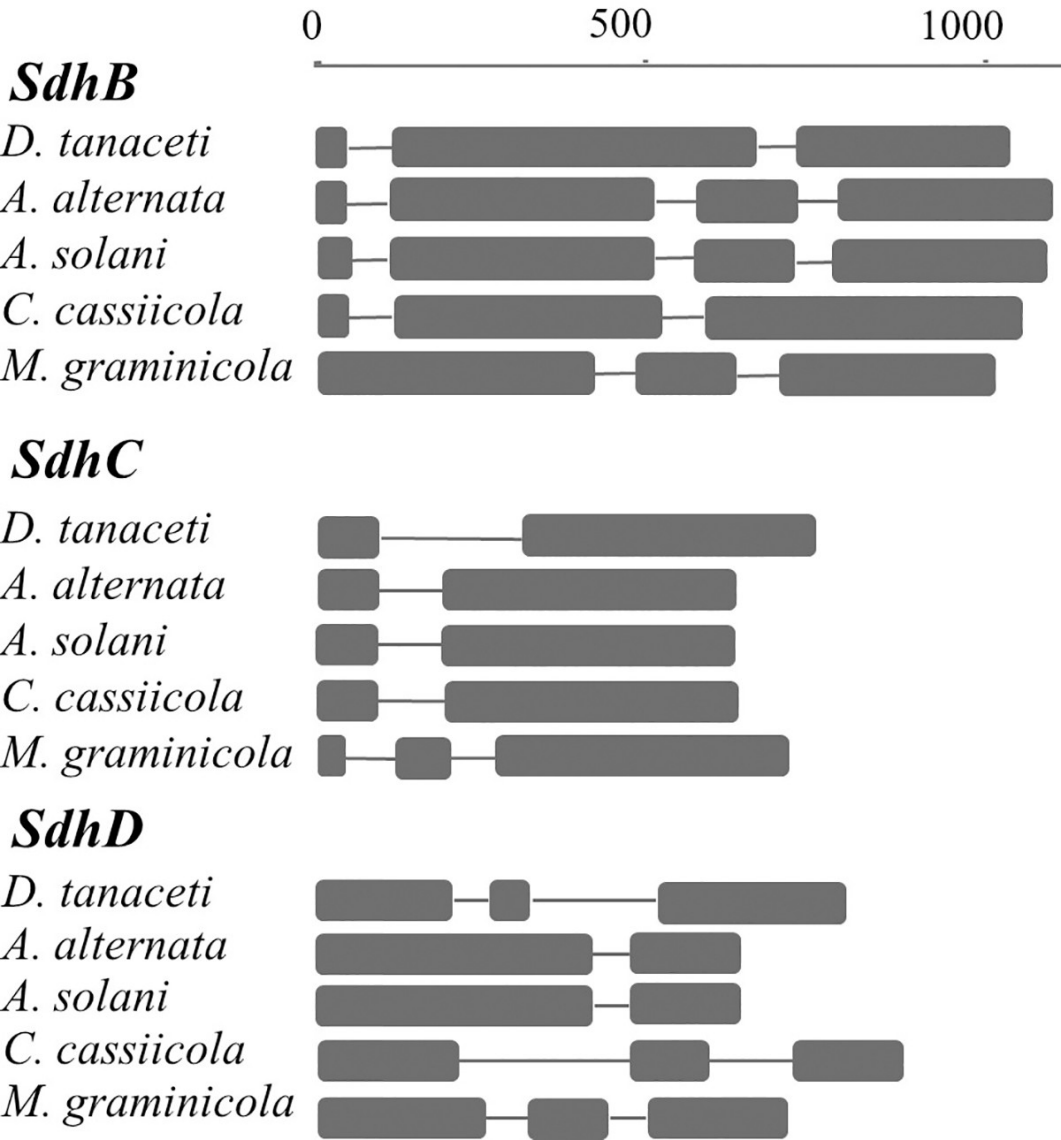
**Structural arrangement of the succinate dehydrogenase subunit B, C and D genes of *Didymella tanaceti*, *Alternaria alternata* strain AR-SBL-4S [26], *A*. *solani* strain 1178-W1 [27], *Corynespora cassiicola* strain IbCor0008 [25], and *Mycosphaerella graminicola* strain R39-1 [16].** Squares indicate exons. Lines connecting the squares indicate introns. The scale bar indicates gene length (base pair).

**Fig 2:**
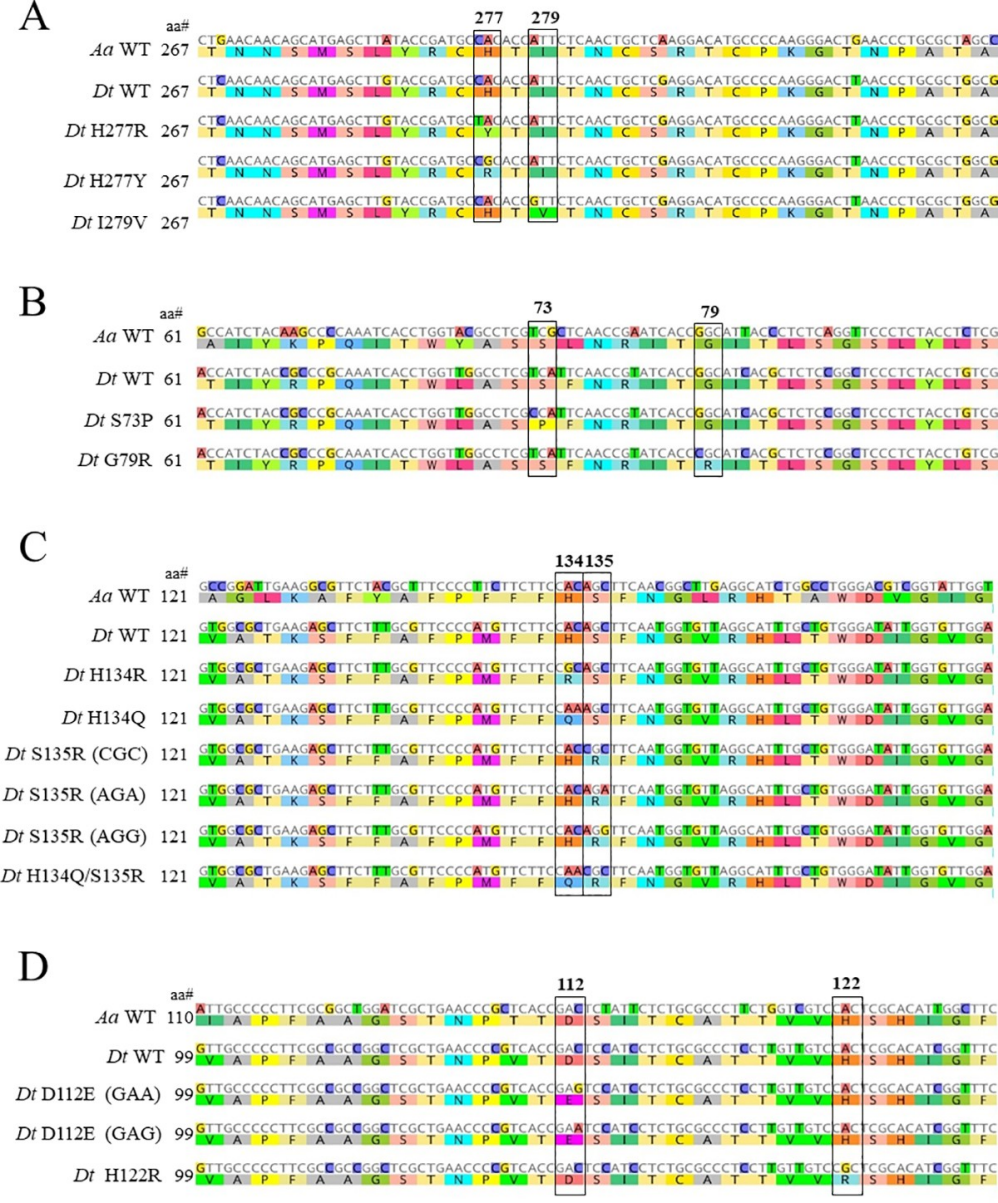
**MAFFT alignment of partial succinate dehydrogenase gene sequences found in *Didymella tanaceti* (*Dt*) wild type (WT) and mutant isolates with WT sequences of *Alternaria alternata* (*Aa*) strain AR-SBL-4S [26] for (A) *Sdh*B, (B,C) *Sdh*C and (D) *Sdh*D.** Reference amino acid numbers relative to each species are located to the left of the alignments. Black boxes/coloured regions indicate the amino acid substitutions found in *D*. *tanaceti*.

The predicted *Sdh*B of *D*. *tanaceti* was 1,034-base pairs (bp) and encoded 306 amino acids (aa). The opening reading frame (ORF) was arranged into three exons, with two putative introns of 61 and 52-bp in length (Fig 1). Species sequence alignment identified that intron splice sites were identical to *A*. *alternata*, except for an intron located 6-bp upstream from the first conserved cysteine region in *A*. *alternata*, which was absent in *D*. *tanaceti*. This intron also occurred in *A*. *solani*, *Zymoseptoria tritici* (syn: *Mycosphaerella graminicola*) and *Corynespora cassiicola* (Fig 1). Comparison of the encoded SdhB protein sequence of *D*. *tanaceti* with other fungal species showed high amino acid conservation of the three cysteine rich clusters, associated with the iron-sulphur centres. MAFFT alignment of the SdhB protein of *D*. *tanaceti* isolates identified two polymorphic codons located within the third conserved cysteine region (Fig 2A). Histidine (CAC) to tyrosine (TAC) or arginine (CGC) substitutions occurred at codon 277 (H277Y/R) in 16 and 6 isolates, respectively. At codon 279 an isoleucine (ATT) to valine (GTT) substitution (I279V) occurred in one isolate (Fig 2A). In addition, the fourteenth nucleotide of the second intron of 15 *D*. *tanaceti* isolates exhibited an adenine (A) to guanine (G) transition.

The predicted *Sdh*C of *D*. *tanaceti* was 746-bp and encoded 177-aa. The ORF was arranged into two exons, with a putative intron of 212-bp (Fig 1). The intron splice site was identical to *A*. *alternata*, *A*. *solani* and *C*. *cassiicola*, though the intron length was more than double in *D*. *tanaceti* (Fig 1). MAFFT alignment of the encoded SdhC protein of *D*. *tanaceti* isolates identified four polymorphic amino acid residues (Fig 2B and 2C). The most common of these was a histidine (CAC) to arginine (CGC) substitution which occurred at codon 134 (H134R) in eight isolates. At codon 88, a leucine (CTG) to methionine (ATG) substitution (L88M) occurred in four isolates. Single isolates containing either a glycine (GGC) to arginine (CGC) substitution at codon 79 (G79R) or a serine (AGC) to arginine (AGG) substitution at codon 135 (S135R) were observed (Fig 2B and 2C). A single-nucleotide polymorphism (SNP) (C/T) resulting in a synonymous substitution was also identified at codon 32 in four isolates. In addition, two G/A SNPs were identified within the putative intron. One isolate contained compound substitutions of L88M and H134R.

The predicted *Sdh*D of *D*. *tanaceti* was 784-bp and encoded 182-aa. The ORF was arranged into three exons, with two putative introns of 50 and 185-bp (Fig 1). The first intron in *D*. *tanaceti* was not present in *A*. *alternata*, *A*. *solani*, *Z*. *tritici* and *C*. *cassiicola*, while the splice site for the second intron were identical in *D*. *tanaceti*, *Z*. *tritici* and *C*. *cassiicola*. MAFFT alignment of the SdhD protein of *D*. *tanaceti* isolates identified two polymorphic amino acid residues. An aspartate (GAC) to glutamic acid (GAG or GAA) substitution occurred at codon 112 (D112E) in three isolates and a histidine (CAC) to arginine (CGC) substitution occurred at codon 122 (H122R) in one isolate (Fig 2D). A SNP (C/T) resulting in a synonymous substitution was also identified at codon 45. In addition, three SNPs (C/T, G/A and G/A) were identified within the two putative introns. Isolates with compound substitutions at codons 112 and 122 were not found.

Three isolates with compound substitutions in two different Sdh subunits were identified. Each of these isolates contained the SdhC-L88M with either of the SdhD-D112E or SdhB-H277Y. Isolates with no substitution in any of the subunits were considered wild-type (WT). Comparison of the Sdh genotypes identified in this study and the boscalid response phenotype of isolates, previously assigned to them by Hay *et al*. [7], identified that all genetically WT *D*. *tanaceti* isolates and the single SdhB-I279V isolate had a sensitive phenotype[7].

### HRM assay for *Sdh* allele detection

**Fig 3:**
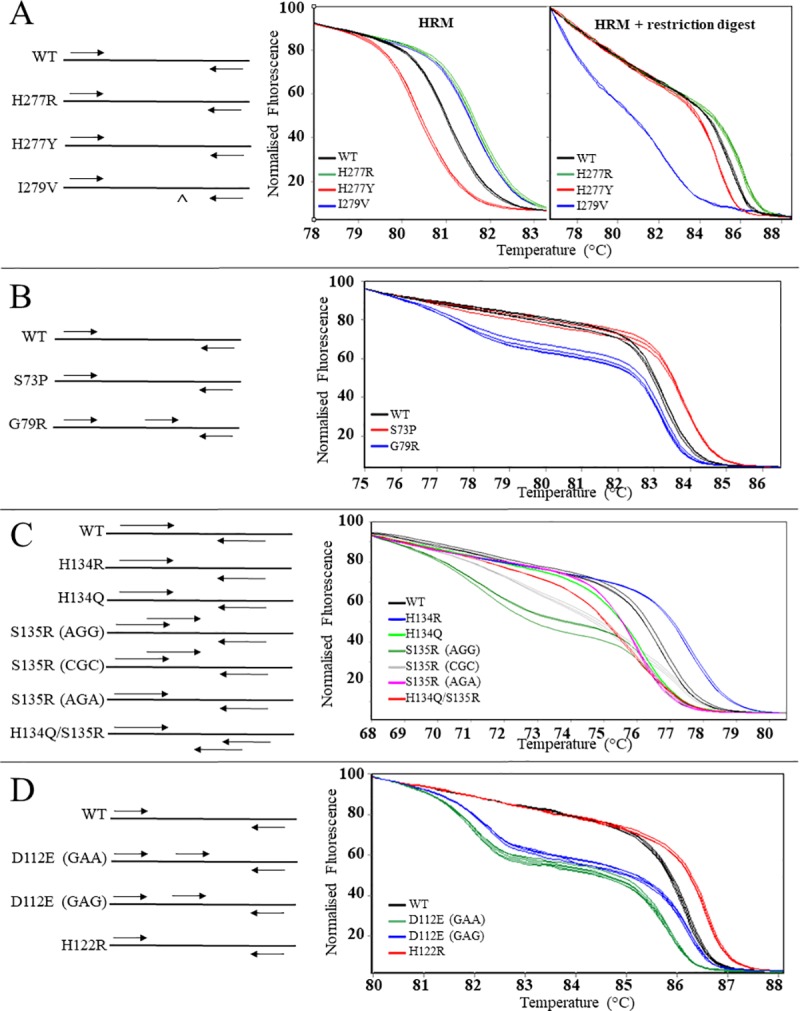
High resolution melt analysis for allele detection in the succinate dehydrogenase (*Sdh*) genes of *Didymella tanaceti*. Left hand side of figure indicates primer binding regions and right-hand side shows normalised fluorescence for each of the known substitutions and wild-type in (A) SdhB codons 277 and 279; (B) SdhC codons 73 and 79; (C) SdhC codons 134 and 135 and (D) SdhD codons 112 and 122. On left hand side, black lines indicate the gene and arrows indicate the annealing position of primers (not to scale).

The developed HRM assays were able to identify the known mutations in each of the *Sdh*B, *Sdh*C and *Sdh*D (Fig 3A–3D). Additional mutations were identified in the initial *Sdh*C HRM screens by the occurrence of unique novel melt curves. The location and nature of these additional mutations was confirmed by gene sequencing. A serine (TCA) to proline (CCA) substitution occurred at codon 73 (S73P), a histidine (CAC) to glutamine (CAA) occurred at codon 134 and a combined histidine (CAC) to glutamine (CAA) and serine (AGC) to arginine (CGC) occurred at codons 134 and 135 (H134Q/S135R) (Fig 2B and 2C). Furthermore, two additional mutations (AGC to CGC and AGC to AGA) were identified which also conferred substitution of serine with arginine at codon 135 (S135R) (Fig 2B and 2C). The SdhC HRM assays were adapted, as required, to identify these additional mutations (Fig 3B and 3C). For all the HRM assays the results from isolates run in duplicate were consistent and *Sdh*B, *Sdh*C and *Sdh*D sequencing results of the 92 *D*. *tanaceti* isolates selected for *in vitro* boscalid testing aligned with the HRM results (Table 1). Sequencing results identified the SdhC-L88M substitution in 2 isolates and in compound substitutions with SdhB-H277R (*n* = 1), SdhB-H277Y (*n* = 1), SdhC-G79R (*n* = 1), SdhC-H134R (*n* = 2), SdhC-S135R (*n* = 1), and SdhD-D112E (*n* = 3) isolates.

**Table 1:** EC_50_, resistance factor and associated phenotype of *Didymella tanaceti* isolates (*n* = 92) with known SDH substitutions.

Isolate^a^	Substitution^b^			Accession number	EC_50_^c^	RF^d^	Phenotype^e^	Boscalid concentrations tested (μg a.i./mL)
	SdhB	SdhC	SdhD					
041–0002				MK500737- MK500739	0.02	0.5	S	0, 0.001, 0.01, 0.05, 0.25, 0.5, 1.25
041–0032					0.04	0.9	S	0, 0.001, 0.01, 0.05, 0.25, 0.5, 1.25
041–0034					0.05	1.3	S	0, 0.001, 0.01, 0.05, 0.25, 0.5, 1.25
041–0047		L88M		MK500740- MK500743	0.04	0.9	S	0, 0.001, 0.01, 0.05, 0.25, 0.5, 1.25
041–0049					0.09	2.2	S	0, 0.001, 0.01, 0.05, 0.25, 0.5, 1.25
041–0076					0.04	1.0	S	0, 0.001, 0.01, 0.05, 0.25, 0.5, 1.25
041–0077		L88M			0.04	0.9	S	0, 0.001, 0.01, 0.05, 0.25, 0.5, 1.25
041–0231					0.04	0.9	S	0, 0.001, 0.01, 0.05, 0.25, 0.5, 1.25
F50607A					0.03	0.8	S	0, 0.001, 0.01, 0.05, 0.25, 0.5, 1.25
F50607B					0.03	0.7	S	0, 0.001, 0.01, 0.05, 0.25, 0.5, 1.25
041–0001	H277Y			MK500757	27.60	673.2	HR	0, 2.5, 5, 10, 25, 50
041–0004	H277Y	L88M		MK500758	15.90	387.8	HR	0, 2.5, 5, 10, 25, 50
041–0009	H277Y				19.80	482.9	HR	0, 2.5, 5, 10, 25, 50
041–0015	H277Y				19.40	473.2	HR	0, 2.5, 5, 10, 25, 50
041–0022	H277Y				50.00	1219.5	VHR	0, 2.5, 5, 10, 25, 50
041–0041	H277Y				15.00	365.9	HR	0, 2.5, 5, 10, 25, 50
041–0088	H277Y			MK500760	17.30	422.0	HR	0, 2.5, 5, 10, 25, 50
041–0242	H277Y				12.90	314.6	HR	0, 2.5, 5, 10, 25, 50
041–1335	H277Y				2.87	70.0	MR	0, 2.5, 5, 10, 25, 50
041–1338	H277Y				12.70	309.8	HR	0, 2.5, 5, 10, 25, 50
041–0018	H277R				9.17	223.7	HR	0, 2.5, 5, 10, 25, 50
041–0024	H277R			MK500759	9.80	239.0	HR	0, 2.5, 5, 10, 25, 50
041–0031	H277R				5.41	132.0	HR	0, 2.5, 5, 10, 25, 50
041–0252	H277R				2.56	62.4	MR	0, 2.5, 5, 10, 25, 50
041–0414	H277R				8.28	202.0	HR	0, 2.5, 5, 10, 25, 50
041–0485	H277R				10.40	253.7	HR	0, 2.5, 5, 10, 25, 50
041–0586	H277R				8.63	210.5	HR	0, 2.5, 5, 10, 25, 50
041–0744	H277R				15.60	380.5	HR	0, 2.5, 5, 10, 25, 50
041–0748	H277R	L88M		MK500762	11.20	273.2	HR	0, 2.5, 5, 10, 25, 50
041–1641	H277R				10.20	248.8	HR	0, 2.5, 5, 10, 25, 50
041–0039	I279V				0.90	21.9	MR	0, 0.05, 0.25, 0.5, 1.25, 2.5
041–0227	I279V			MK500761	0.38	9.3	LR	0, 0.05, 0.25, 0.5, 1.25, 2.5
041–0235	I279V				0.37	9.1	LR	0, 0.05, 0.25, 0.5, 1.25, 2.5
041–0253	I279V				0.48	11.8	MR	0, 0.05, 0.25, 0.5, 1.25, 2.5
041–0254	I279V				0.33	8.1	LR	0, 0.05, 0.25, 0.5, 1.25, 2.5
041–0267	I279V				1.87	45.6	MR	0, 0.05, 0.25, 0.5, 1.25, 2.5
041–0766	I279V				0.59	14.4	MR	0, 0.05, 0.25, 0.5, 1.25, 2.5
041–0902	I279V				0.45	11.0	MR	0, 0.05, 0.25, 0.5, 1.25, 2.5
041–0914	I279V				0.52	12.8	MR	0, 0.05, 0.25, 0.5, 1.25, 2.5
041–0136		S73P		MK500751	19.90	485.4	HR	0, 0.5, 5, 10, 25, 50
041–0958		S73P			7.95	193.9	HR	0, 0.5, 5, 10, 25, 50
041–0989		S73P			6.22	151.7	HR	0, 0.5, 5, 10, 25, 50
041–0992		S73P			6.76	164.9	HR	0, 0.5, 5, 10, 25, 50
041–1029		S73P			20.90	509.8	HR	0, 0.5, 5, 10, 25, 50
041–0204		G79R		MK500753	30.00	731.7	HR	0, 0.5, 5, 10, 25, 50
041–0245		G79R			31.50	768.3	HR	0, 0.5, 5, 10, 25, 50
041–0259		G79R			30.90	753.7	HR	0, 0.5, 5, 10, 25, 50
041–0814		G79R/L88M		MK500756	46.10	>1000	VHR	0, 0.5, 5, 10, 25, 50
041–0984		G79R			43.60	>1000	VHR	0, 0.5, 5, 10, 25, 50
041–1039		G79R			23.90	582.9	HR	0, 0.5, 5, 10, 25, 50
041–1298		G79R			29.20	712.2	HR	0, 0.5, 5, 10, 25, 50
CF196		G79R			21.30	519.5	HR	0, 0.5, 5, 10, 25, 50
041–0007		H134R		MK500748	>50	>1000	VHR	0, 2.5, 5, 10, 25, 50
041–0016		H134R/L88M		MK500749	>50	>1000	VHR	0, 2.5, 5, 10, 25, 50
041–0025		H134R			42.20	1029.3	VHR	0, 2.5, 5, 10, 25, 50
041–0027		H134R			>50	>1000	VHR	0, 2.5, 5, 10, 25, 50
041–0107		H134R			>50	>1000	VHR	0, 2.5, 5, 10, 25, 50
041–0188		H134R/L88M			>50	>1000	VHR	0, 2.5, 5, 10, 25, 50
041–0522		H134R			35.30	861.0	HR	0, 2.5, 5, 10, 25, 50
041–0622		H134R			16.60	404.9	HR	0, 2.5, 5, 10, 25, 50
041–1255		H134R			>50	>1000	VHR	0, 2.5, 5, 10, 25, 50
041–1345		H134R			>50	>1000	VHR	0, 2.5, 5, 10, 25, 50
041–0391		H134Q		MK500754	3.0	71.9	MR	0, 2.5, 5, 10, 25, 50
041–0162		S135R		MK500752	26.2	639.0	HR	0, 2.5, 5, 10, 25, 50
041–0328		S135R			16.2	395.1	HR	0, 2.5, 5, 10, 25, 50
041–0358		S135R			13.5	329.3	HR	0, 2.5, 5, 10, 25, 50
041–0370		S135R			25.6	624.4	HR	0, 2.5, 5, 10, 25, 50
041–0397		S135R			25.1	612.2	HR	0, 2.5, 5, 10, 25, 50
041–0455		S135R			13.4	326.8	HR	0, 2.5, 5, 10, 25, 50
041–0784		S135R/L88M		MK500755	>50	>1000	VHR	0, 2.5, 5, 10, 25, 50
041–1344		S135R			13.5	329.3	HR	0, 2.5, 5, 10, 25, 50
041–1350		S135R			29.4	717.1	HR	0, 2.5, 5, 10, 25, 50
CF2		S135R			32.9	802.4	HR	0, 2.5, 5, 10, 25, 50
041–0132		H134Q/S135R		MK500750	24.30	592.7	HR	0, 2.5, 5, 10, 25, 50
041–0163		H134Q/S135R			26.70	651.2	HR	0, 2.5, 5, 10, 25, 50
041–0193		H134Q/S135R			25.50	622.0	HR	0, 2.5, 5, 10, 25, 50
041–0201		H134Q/S135R			23.60	575.6	HR	0, 2.5, 5, 10, 25, 50
041–0875		H134Q/S135R			16.40	400.0	HR	0, 2.5, 5, 10, 25, 50
041–0035			D112E	MK500743	5.52	134.6	HR	0, 0.5, 1.25, 2.5, 5, 10
041–0036		L88M	D112E	MK500744	4.88	119.0	HR	0, 0.5, 1.25, 2.5, 5, 10
041–0038		L88M	D112E	MK500745	2.80	68.3	MR	0, 0.5, 1.25, 2.5, 5, 10
041–0335			D112E		4.63	112.9	HR	0, 0.5, 1.25, 2.5, 5, 10
041–0340			D112E		9.22	224.9	HR	0, 0.5, 1.25, 2.5, 5, 10
041–0435		L88M	D112E	MK500747	3.56	86.8	MR	0, 0.5, 1.25, 2.5, 5, 10
041–0439			D112E		2.82	68.8	MR	0, 0.5, 1.25, 2.5, 5, 10
041–1282			D112E		1.88	45.9	MR	0, 0.5, 1.25, 2.5, 5, 10
041–1333			D112E		1.30	31.7	MR	0, 0.5, 1.25, 2.5, 5, 10
F50403B			D112E		3.02	73.7	MR	0, 0.5, 1.25, 2.5, 5, 10
041–0297			H122R	MK500746	9.29	226.6	HR	0, 0.5, 5, 10, 25, 50
041–1111			H122R		32.20	785.4	HR	0, 0.5, 5, 10, 25, 50
041–1130			H122R		21.00	512.2	HR	0, 0.5, 5, 10, 25, 50
CF149			H122R		39.10	953.7	HR	0, 0.5, 5, 10, 25, 50

^a^ *Didymella tanaceti* isolates stored in the Tasmanian Institute of Agriculture fungal collection, Tasmania, Australia. All isolates originally recovered from pyrethrum leaves.

^b^ Substitution identified via high resolution melt assay and confirmed by sequencing.

^c^ EC_50_ = estimated boscalid concentration (μg/a.i./mL) to reduce radial growth by 50% of the non-amended controls. Calculated using logistic regression of the relative growth against log_10_ of the boscalid concentration.

^d^ Resistance Factor (RF) =  EC_50_X/EC_50_WT, where EC_50_X is the EC_50_ value of the isolate being examined, and EC_50_WT is the average EC_50_ value of the baseline isolates (WT).

^e^ Boscalid phenotype based on the interpretation of RF values; S = sensitive (RF ≤ 3) LR = low resistance (3 > RF ≤ 10), MR = moderate resistance (10 > RF ≤ 100), HR = high resistance (100 > RF ≤ 1000) and VHR = very high resistance (RF >1000).

### *In vitro* boscalid testing

Within the *D*. *tanaceti* isolates selected for *in vitro* boscalid testing (*n* **=** 92; Table 1), substitutions were associated with varying resistant phenotypes. Wild type isolates (baseline isolates; *n* = 10) had EC_50_ values ranging from 0.02 to 0.09 μg a.i/mL (average = 0.041 μg a.i/mL), resulting in a resistance factor (RF) ≤ 2.2 and a sensitive phenotype (Table 1). Of the nine isolates exhibiting a SdhB-I279V substitution, three exhibited low resistance to boscalid (EC_50_ = 0.33–0.38 μg a.i./mL) and six were moderately resistant (EC_50_ = 0.45–1.87) (Table 1). Of the ten isolates containing a SdhB-H277Y substitution, eight were highly resistant (EC_50_ = 12.70–27.60), one was moderately resistant (EC_50_ = 2.87) and one exhibited very high resistance (EC_50_ = 50.0; Table 1). Of the ten isolates exhibiting the other SdhB substitution at codon 277, SdhB-H277R, nine were highly resistant (EC_50_ = 5.40–15.60) with the other moderately resistant (EC_50_ = 2.56). All substitutions in the SdhC resulted in isolates with high or very high resistance. All five isolates with a SdhC-S73P substitution were highly resistant (EC_50_ = 6.20–20.90). Isolates with the SdhC-G79R substitution, had either high resistance (*n* = 6; EC_50_ = 21.30–31.50) or very high resistance (*n =* 2; EC_50_ = 43.60–46.10). The highest resistance to boscalid was identified in the ten isolates with the SdhC-H134R substitution with eight exhibiting very high resistance (EC_50_ 42.20 –>50.0) and the remainder high resistance (EC_50_ = 16.60–35.30). The single isolate with a SdhC-H134Q substitution was moderately resistant (EC_50_ = 3.0). Of the ten isolates with the SdhC-S135R substitution in the adjacent codon, nine were highly resistant (EC_50_ 13.50–32.90) while one isolate exhibited very high resistance (EC_50_ > 50.0). The five isolates with combined SdhC-H134Q/S135R substitutions were all highly resistant (EC_50_ = 16.4–26.7). The two isolates containing the SdhC-L88M substitution in solitary were as sensitive to boscalid as WT isolates. The occurrence of the SdhC-L88M substitution did not affect the fungicide response of the nine isolates containing it in combination with another substitution. Therefore, it was concluded that the SdhC-L88M substitution was not associated with any additional resistance even when in combination with other substitutions. All substitutions in the SdhD resulted in isolates with moderate or high resistance (Table 1). Of the ten isolates with a SdhD-D112E substitution, six were moderately resistant (EC_50_ = 1.30–3.60) and the remainder highly resistant (EC_50_ = 4.60–9.20). Furthermore, all four isolates with a SdhD-H122R substitution exhibited high resistance (EC_50_ = 9.29–39.10).

### Frequency and genetic diversity of Sdh substitutions in the 2012 *D*. *tanaceti* population

In August 2012, prior to application of the spring fungicide program, 92.9% of *D*. *tanaceti* isolates from the four 2012 field populations contained a substitution associated with decreased sensitivity in the SdhB, SdhC or SdhD (Table 2). No isolates containing compound substitutions of interest were identified. Following application of the spring fungicide program the percent of isolates sampled in November 2012 containing a Sdh substitution increased to 98.9% (Table 2). This result coincided with no detection of WT isolates in three of the four fields (Table 2). The SdhB-H277Y substitution was the most common Sdh substitution in each field, irrespective of sampling period. Multinomial logistic regression of genotype frequency indicated a significant interaction between field and time of sampling (*P* = 0.0065). Post-hoc pairwise comparisons between sampling periods within fields and substitution found that significant reductions in WT frequency occurred from August to November in fields 12–63242 and 12–70046 (Table 2). Significant increases (*P* < 0.05) in Sdh substitution frequency within fields were observed for SdhB-H277R (Field 12–51907) and SdhC-S73P (Field 12–70047) from August to November (Table 2). Conversely, significant decreases (*P* < 0.05) over the same period within fields were observed for SdhC-H134R (Field 12–51097) and SdhD-D112E (Field 12–70046). A single isolate with the SdhC-H134Q substitution was identified across all fields and sampling periods. This substitution and three other substitution occurring at frequencies less than 1% were excluded from the analysis to prevent detrimental effects on model estimates (Table 2).

**Table 2:** Frequency (%) of each SDH substitution in the 2012 *Didymella tanaceti* population by sampling period and field.

Subunit-substitution	12–51907		12–63242		12–70046		12–70047		All Fields		Overall
	Aug^a^	Nov^a^	Aug	Nov	Aug	Nov	Aug	Nov	Aug	Nov	
Wildtype	5.4	3.3	**16.2**^**b**^	**0.0**	**11.6**	**0.0**	1.0	0.0	**7.1**	**1.1**	3.6
SdhB-H277R	**4.5**	**16.4**	5.9	17.2	9.3	11.9	10.8	7.2	**7.4**	**12.5**	10.3
SdhB-H277Y	58.0	59.9	38.2	48.4	46.5	59.7	57.8	64.5	52.3	59.9	56.7
SdhB-I279V	0.0	0.7	7.4	3.1	0.0	0.0	0.0	0.0	1.5	0.7	1.0
SdhC-G79P	0.0	0.7	4.4	0.0	0.0	1.5	0.0	0.6	0.9	0.7	0.8
SdhC-H134Q/S135R	2.7	0.7	1.5	0.0	0.0	0.0	0.0	0.0	1.2	0.2	0.6
SdhC-H134R	**21.4**	**11.8**	23.5	29.7	11.6	20.9	18.6	19.3	19.7	18.5	19.0
SdhC-H134Q	0.0	0.0	0.0	0.0	0.0	0.0	1.0	0.0	0.3	0.0	0.1
SdhC-S135R	1.8	2.0	0.0	0.0	7.0	0.0	3.9	3.0	2.8	1.8	2.2
SdhC-S73P	0.9	0.0	0.0	0.0	0.0	4.5	**0.0**	**2.4**	**0.3**	**1.6**	1.0
SdhD-D112E	5.4	4.6	2.9	1.6	**14.0**	**1.5**	5.9	1.8	**6.2**	**2.7**	4.1
SdhD-H122R	0.0	0.0	0.0	0.0	0.0	0.0	1.0	1.2	0.3	0.4	0.4
Samples^c^	112	152	68	64	43	67	102	166	325	449	774

^a^ Sampling period; Aug = August 2012, Nov = November 2012.

^b^ Sampling period pairs in bold indicate significant (*P <* 0.05) frequency variations between periods based on multinomial logistic regression.

^c^ Total number of individuals observed

Following combination of the microsatellite (SSR) profile and mating-type (MAT) of isolates in the 2012 *D*. *tanaceti* population, 138 genetically unique groups (Multi-Locus-Genotypes; MLG) were identified by Pearce *et al*. [28]. Overlaying the Sdh data with the SSR and MAT profile identified that no Sdh substitution observed in multiple individuals was associated with only a single MLG (Table 3). Furthermore, each MLG consisted of isolates containing one to eight different Sdh substitutions. Of the 70 MLGs consisting of ≥ 2 isolates, 48 MLGs were associated with > 1 Sdh substitution (average substitutions per MLG = 2.4). The most frequent substitutions, SdhB-H277Y and SdhC-H134R, were identified in 96 and 46 MLGs, respectively (Table 3). Despite occurring at a low frequency (< 1.0%), all isolates containing the SdhC-G79R and SdhD-H122R substitutions were genetically unique. When the Sdh substitutions were combined with the previously identified MLGs, 236 genetically unique groups were identified (Table 3). The 2012 population dataset is available at 10.6084/m9.figshare.7800929.

**Table 3:** Number of observations of each substitution (*N*) and associated multilocus genotypes (MLGs) broken down by sampling period, including the number shared between the two in the 2012 *Didymella tanaceti* population.

Subunit-substitution	Overall		August		November		Shared MLGs^b^
	*N*	MLGs^a^	*N*	MLGs^a^	*N*	MLGs^a^	
Wild Type	28	21	23	16	5	5	0
SdhB-H277R	80	32	24	15	56	27	10
SdhB-H277Y	439	96	170	67	269	64	35
SdhB-I279V	8	3	5	2	3	2	1
SdhC-S73P	8	3	1	1	7	2	0
SdhC-G79R	6	6	3	3	3	3	0
SdhC-H134R	147	46	64	29	83	36	19
SdhC-H134Q	1	1	1	1	0	0	0
SdhC-S135R	17	13	9	6	8	7	0
SdhC-H134Q/S135R	5	2	4	2	1	1	1
SdhD-D112E	32	10	20	9	12	6	5
SdhD-H122R	3	3	1	1	2	2	0
**Total**	**774**	**236**	**325**	**99**	**449**	**98**	

^a^ Multi-locus genotype derived from combining microsatellite and mating-type data [28]

^b^ Number of multi-locus genotypes found in both sampling periods.

A breakdown of the MLGs associated with each substitution sampled in August and November identified that the WT, SdhC-S73P, SdhC-G79R, SdhC-H134Q, SdhC-S135R and SdhD-H122R isolates obtained in November were genetically different (based on SSR and MAT profile) to those occurring in August, with no MLGs shared between the two sampling periods for each substitution (Table 3). For the SdhB-H277Y and SdhC-H134R isolates only 52.2 and 65.5%, respectively, of the MLGs identified in August were present in November despite a similar number of MLGs identified in each sampling period (Table 3).

## Discussion

Hay *et al*. [7] provided evidence that a decreased sensitivity to boscalid had developed in *D*. *tanaceti* from Tasmanian pyrethrum crops. We report that the observed resistant phenotypes are associated with mutations in the *Sdh*B, *Sdh*C, and *Sdh*D subunits of the succinate dehydrogenase. Sixteen point mutations in gene sequences were detected, resulting in three substitutions in the SdhB, five substitutions in the SdhC and two substitutions in the SdhD. To our knowledge this the largest number of Sdh substitutions recovered in a solitary fungal species on one host associated with resistance. Furthermore, this study provided the first quantifiable estimates of the frequency of *D*. *tanaceti* isolates with decreased boscalid sensitivity in commercial pyrethrum fields.

Isolates with WT Sdh genotypes were sensitive to boscalid indicating this fungicide had provided sufficient control of *D*. *tanaceti* prior to the selection of resistant isolates. This also agrees with field observations following initial introduction of boscalid to the pyrethrum fungicide program. The baseline EC_50_ of the WT isolates established a reference point that indicates sensitivity to boscalid [29, 30]. Wild-type isolates had EC_50_ values less than 0.05 μg a.i./mL. Therefore, the range of 0–0.5 μg a.i./mL used by Hay *et al*. [7] to identify sensitive isolates may have underestimated the true shift in sensitivity of *D*. *tanaceti*. For example, based on the sensitivity range reported by Hay *et al*. [7] isolates with the SdhB-I279V would have been classed as sensitive to boscalid, whereas this study showed using RF, SdhB-I279V isolates were moderately resistant to boscalid. Many fungicide studies do not calculate baseline responses of WT isolates or isolates collected prior to the introduction of the fungicide, but instead infer resistance phenotypes on defined EC_50_ values/ranges [10, 31]. The calculation of a baseline response and subsequent RF allows greater comparison and evaluation of the response to a fungicide among different species and those collected from different hosts, and for establishment of discriminatory dose ranges for detecting major shifts in fungicide sensitivity [20, 32, 33].

Mutations in the third highly-conserved cysteine rich region of the *SdhB* were identified in 70.6% of the 2012 collected *D*. *tanaceti* isolates. The SdhB-H277Y was the most frequently recovered substitution and was associated with high resistance to boscalid. The recovery frequency of this substitution increased from August to November following spring fungicide applications incorporating boscalid in all fields. An additional substitution at the same codon, SdhB-H277R, also associated with high resistance, was found in a further 10% of individuals. Substitutions at homologous positions of these two substitutions in the SdhB were the first to be associated with SDHI resistance in *Z*. *tritici* [34], *B*. *cinerea* [35] and *A*. *alternata* [36]. Furthermore, genetic transformations studies have confirmed that substitutions at homologous positions of the SdhB-H277Y/R substitutions in *Sclerotinia homoeocarpa* and *B*. *cinerea* were determinants of decreased boscalid sensitivity [37, 38]. For many species, including *A*. *alternata* [36], *B*. *cinerea* [39–41], *C*. *cassiicola* [25], and *D*. *bryoniae* [10], the SdhB-H277Y/R are the most frequent substitutions identified from field surveys.

The high proportion of *D*. *tanaceti* isolates with SdhB-H277Y in the present study is not surprising. Lalève *et al*. [42] identified that homolog recombinant mutants of *B*. *cinerea* with SdhB-H272Y (corresponding to SdhB-H277Y in *D*. *tanaceti*) did not show decreased respiration or Sdh activity when compared to the WT strain. In contrast, these SdhB-H272Y transformants displayed higher initial growth than the WT strain on minimal medium at low temperature and in competition assays, in the absence of a selection pressure, the SdhB-H272Y transformants had a competitive advantage over the WT strain with increases from 40% to 70% of population after seven cycles on artificial media *in vitro*. No fitness penalties were found in *A*. *alternata* isolates with the SdhB-H277Y substitution [43]. If a similar lack of fitness cost is associated with SdhB-H277Y in *D*. *tanaceti*, coupled with a decreased sensitivity to boscalid, this may explain its dominance in field populations.

The only other substitution observed in the SdhB of *D*. *tanaceti* was SdhB-I279V. This substitution has also been identified in a single *Stagonosporopsis citrulli* isolate collected from watermelon [44]. The isolate was reported to be sensitive to boscalid and resistant to fluopyram. A substitution corresponding to SdhB-I279V has also been induced in laboratory studies in isolates of *Z*. *tritici* following UV mutagenesis and selection on media [15, 16, 45]. This substitution was associated with RF indicating low to moderate resistance to boscalid in *Z*. *tritici* (RF: 4.2–12.7) [15, 45]. Which is consistent with our findings in *D*. *tanaceti*. Additional substitutions at codon 272 in *B*. *cinerea* (SdhB-H272L and SdhB-H272V), which are associated with high resistance to boscalid [46], have not been observed in *D*. *tanaceti* to date.

Two substitutions at codon 134 of SdhC were identified in *D*. *tanaceti* with varied boscalid responses. SdhC-H134R isolates exhibited very high resistance levels, while the SdhC-H134Q exhibited moderate resistance levels. The SdhC-H134Q was identified in only one *D*. *tanaceti* isolate, though it was found in combination with SdhC-S135R in five isolates. This substitution was identified in *A*. *solani* isolates from potato in Germany in 2014 [47]. In contrast to our studies, *in vitro* EC_50_ values for the *A*. *solani* SdhC-H134Q isolates were above 100 μg boscalid/mL [47]. The SdhC-H134R was found in 19% of the 2012 *D*. *tanaceti* population and did not significantly change in frequency between the two sampling periods in three of four fields sampled. However, in the fourth field, frequency decreased nearly 50% from August to November. Fitness studies in *A*. *alternata* by Fan *et al*. [43] found that SdhC-H134R isolates remained competitive against sensitive isolates over the course of five successive transfers in the absence of fungicides. At the neighbouring codon, 135, three alternative point mutations encoded the SdhC-S135R substitution. Both Sierotzki *et al*. [48] and Lichtemberg *et al*. [49] have reported on the presence of SdhC-S135R in *A*. *alternata* isolate from pistachio as exhibiting resistance to carboxamides. This substitution was also reported to occur in *P*. *teres* on winter barley from Germany in 2013 [50]. As in our study, in these cases, the frequency of occurrence was low (0.6–2%). *D*. *tanaceti* isolates with the combined SdhC-H134Q/S135R had high resistance profiles, more consistent with the isolates with only the SdhC-S135R than the single tested SdhC-H134Q isolate. An association between the SdhC-L88M substitution and decreased boscalid sensitivity was not identified. These results suggest that this substitution does not alter the boscalid binding site in the succinate dehydrogenase protein.

The remaining SdhC substitutions identified in *D*. *tanaceti* were only found at a low frequency. The SdhC-S73P substitution has been identified in *C*. *cassiicola* in cucumber in Japan and was associated with moderate resistance to boscalid [25]. This substitution is homologous in position to an alanine to valine substitution at codon 84 (A84V) induced in a laboratory mutant of *Z*. *tritici* [45]. While inferring low resistance (RF = 1.7) to boscalid the *Z*. *tritici* mutant was highly resistance to other SDHI fungicides. The SdhC-G79R substitution has been identified in *A*. *alternata* isolates in peach in South Carolina [51] and in *Pyrenophora teres* isolates in barley in Europe [50]. In contrast to *D*. *tanaceti*, for *P*. *teres* this was the most frequent mutation identified in isolates from barley and was associated with moderate resistance to boscalid [50]. A substitution at a homologous position to G79R has been detected in laboratory strains of *Z*. *tritici* (G90R) but not in field samples [52, 53]. All the SdhC-G79R *D*. *tanaceti* isolates were genetically unique, indicating either multiple independent origins, recombination of the substitution into genetic diverse individuals, or a pathogen that has a high mutation frequency.

The SdhD-D112E substitution was encoded by two different point mutations in *D*. *tanaceti* and occurred in 4% of the 2012 population. An homologous substitution (SdhD-D133E) have also been found in *A*. *alternata* and *A*. *solani* [27, 47]. Fitness testing of D133E isolates of *A*. *alternata* from peach indicated that whilst having rapid mycelial growth, isolates were hypersensitive to oxidative stress [43], which may explain the lower prevalence of this genotype in the *D*. *tanaceti* population. A second SdhD substitution could only be found in four *D*. *tanaceti* isolates (SdhD-H122R), but homologous substitutions are also reported in *S*. *sclerotiorum* (H132R) [35, 54], *B*. *cinerea* (H132R) [39], *A*. *solani* (H133R) [27] and *A*. *alternata* (H133R) [55].

The inclusion of the SSR and mating-type data in this study provides an insight into the development of resistance within the pathogen population. The results suggested that resistance in *D*. *tanaceti* has not developed due to selection pressure favouring clonal build-up of resistant individuals. Rather, resistance is associated with a wide range of genotypes. The development of fungicide resistance in populations may have varying associations with population genetic structure. For example, Knapova and Gisi [56] found no association between SSR genotype and sensitivity to phenylamide in *Phytophthora infestans*, while Shrestha *et al*. [57] found that QoI resistant *Cersospora sojina* isolates clustered in a single group and were represented by a dominant clonal lineage. The development of a rapid method for allele detection provides an additional tool to analyse the impact of SDHI fungicide application on the frequency of mutations in *D*. *tanaceti* populations and for the ongoing implications and disease management options to be more greatly assessed. Previously, experiments evaluated fungicides based on infection levels pre- and post-application. However, a fungicide that appears efficacious and results in a decrease in pathogen load may be imposing a selection pressure on resistant individuals. The HRM assay will be utilised to evaluate historical changes in Sdh substitution changes in *D*. *tanaceti* populations from 2004 onwards.

The fields sampled as part of the 2012 population collection were first-harvest crops that had not received an application of boscalid, or any other SDHI fungicide, prior to the first sampling in August. Due to the high level of isolates with a mutation in the *Sdh* genes, associated with a decreased sensitivity to boscalid, it is proposed that these may have been introduced to, rather than produced in these fields. *Didymella tanaceti* can be seed-borne [58, 59]. As a perennial species seed-borne isolates may have been exposed to multiple applications of boscalid over the life of the seed crops and developed resistance prior to seed infection. However, the seed used to sow field 12–51907 was harvested in 2006/2007, and it is assumed that boscalid resistant isolates were undetectable in 2006/2007. Boscalid was first used commercially in pyrethrum crops in 2005. Thus for resistant individuals to be introduced to field 12–51907 via seed, boscalid resistant isolates would have had to be been prevalent 12 months after the introduction of boscalid use in pyrethrum and three years before fungicide control failures were identified by Hay *et al*. [7]. An alternative scenario, is that ascospores or other wind-blown inoculum also allows movement of resistance individuals between fields. Scott *et al*. [59] indicated that fields within 2 km of each other are able to contribute inoculum to each other and a sexual cycle for *D*. *tanaceti* cannot be dismissed [60]. The occurrence of the low frequency mutations in multiple fields further supported this hypothesis. Pyrethrum stubble has also been shown to be an inoculum source of *D*. *tanaceti* (T.L Pearce, Tasmanian Institute of Agriculture, Burnie, Australia, personal communication) and following termination of a pyrethrum crop it has been common practice to reincorporate the remaining plant stubble into the soil. The length of time that burned inoculum can remain viable is currently unknown, but this may also allow between season spread.

The high proportion of boscalid resistant isolates within the 2012 characterised population indicated that boscalid resistance in *D*. *tanaceti* contributed to the rapid increase in the frequency and incidence of tan spot. This resistance has occurred despite implementation of management practices by the pyrethrum industry to limit the development of fungicide resistance, including limiting single season applications and alternating mode of action fungicides. However, other pyrethrum pathogens, including *S*. *tanaceti* exhibit no evidence of a reduced sensitivity to boscalid [7], suggesting that the imposed management strategies are working for some species. Furthermore, it could be argued that *S*. *tanaceti* has greater exposure to and selection pressure imposed from boscalid than *D*. *tanaceti*, as the *S*. *tanaceti* pathogen incidence was higher relative to *D*. *tanaceti* following the period of boscalid introduction [58, 61]. Management of ray blight, caused by *S*. *tanaceti*, was the original justification for the deployment of boscalid in pyrethrum cropping [62]. This suggests that a low proportion of *D*. *tanaceti* already had resistance to SDHI fungicides or that *D*. *tanaceti* has a greater capacity to rapidly mutate to generate fungicide resistance traits. Analysis of *D*. *tanaceti* populations has identified high genetic variation among individuals, inferring a high adaptive ability [28]. Furthermore, while no evidence of a sexual cycle in *S*. *tanaceti* has been found [63], a sexual cycle in *D*. *tanaceti* cannot be dismissed [60] and would allow long range dispersal mechanisms and genetic recombination of resistant genotypes.

In 2015, boscalid was removed from the pyrethrum spring fungicide program due to concerns with control failures. However, other SDHI fungicides are commonly applied for control of tan spot and other disease in pyrethrum As cross resistance to multiple SDHI chemicals is reported for many Sdh mutations [19, 55, 64], examination of the Sdh cross resistance profile of *D*. *tanaceti* would be beneficial in identifying fungicide chemistries which will control individuals with the common SdhB-H277Y and SdhC-H134R substitutions and those that confer high resistance to boscalid. For example, *B*. *cinerea* isolates with the SdhB-H272Y/R substitutions have moderate resistance to both boscalid and penthiopyrad [19], but are sensitive or have low resistance to fluopyram [46, 65] and bixafen [65]. Similar cross-resistance patterns between SDHIs have been found in *D*. *bryoniae* [66] and *A*. *alternata* [55]. The pyrethrum industry remains reliant upon SDHI fungicides in the medium term for the control of fungal pathogens other than *D*. *tanaceti*. It is therefore recommended that alternation of SDHI chemicals across seasons, based knowledge of their cross-resistance profile, coupled with population monitoring for specific mutations be employed to mitigate the effects of fungicide resistance in *D*. *tanaceti*.

## Materials and methods

### *Sdh*B, *Sdh*C and *Sdh*D sequences

To identify if a decreased sensitivity to boscalid was associated with mutations in the *Sdh* gene complex, the complete *Sdh*B, *Sdh*C and *Sdh*D genes of 51 *D*. *tanaceti* isolates of known boscalid response phenotypes [7] were sequenced. This included isolates collected from 2009–2012 during which a decreased sensitivity to boscalid was first identified. The *Sdh*B (GenBank acc: KJ426258), *Sdh*C (GenBank acc: KJ426263) and *Sdh*D (GenBank acc: KJ426268) gene sequences of *A*. *alternata* isolate AR-SBL-4S [26] were utilized as query sequences in a BLASTn search against a genomic database consisting of genome sequence data of *D*. *tanaceti* strain BRIP 61988 [60] in Geneious v7.1 (Biomatters Ltd., Auckland, New Zealand) to identify homologous genes in *D*. *tanaceti*.

DNA was extracted from isolates as described by Pearce *et al*. [28]. Entire *Sdh*B, *Sdh*C and *Sdh*D gene sequences were amplified with primers Dt_SdhB_F/Dt_SdhB_R, Dt_SdhC_F/Dt_SdhC_R and Dt_SdhD_F/Dt_SdhD_R, respectively (Table 4), designed using Primer3 v2.3.4 [67]. PCR reactions were performed in a C1000 thermocycler (BioRad, Hercules, CA) in a total volume of 20 μL. Reaction mixes contained 0.05 U TopTaq polymerase (Qiagen, Hilden, Germany), 1 × PCR Buffer, 1 × CoralLoad, 0.4 μM of each primer, 200 μM of each dNTP (Bioline, Alexandria, Australia) and 3–5 ng of genomic DNA. Conditions for amplification were an initial denaturation of 5 min at 94°C, followed by 30 cycles of denaturation at 94°C for 30 s, annealing at 52°C (*Sdh*B), 55°C (*Sdh*C) or 56°C (*Sdh*D) for 30 s, and extension at 72°C for 1 min, with a final elongation of 72°C for 5 min on the last cycle. Amplified products were visualised via gel electrophoresis using a 1.5% (*w*/*v*) agarose gel pre-stained with GelRed (Biotium Inc., Fremont, CA) in 1 × TAE buffer. PCR products were prepared for sequencing using the UltraClean PCR-clean up kit (Mo Bio Laboratories Inc., Carlsbad, CA), according to the manufacturer’s instructions. Sequencing in both directions was conducted at the Australian Genome Research Facility (AGRF; Melbourne, Australia) using Big Dye Terminator v3.1 chemistry and capillary separation on an AB 3730xl DNA Analyser (Applied Biosystems, Foster City, CA).

**Table 4:** Primers used for amplification of *Sdh*B, *Sdh*C and *Sdh*D genes and High-Resolution Melt (HRM) assay.

Primer	Sequence 5’ - 3’	Use
Dt_SdhB_F	TACGTCAGGCTTTAGAAGGAAGGAG	SdhB gene
Dt_SdhB_R	GCCTAACTCAAAATACCAACTGC	SdhB gene
Dt_SdhC_F	GATACGC CCAAGACATCTACG	SdhC gene
Dt_SdhC_R	TACTCACTCCTCCAAATTCGTG	SdhC gene
Dt_SdhD_F	GCTCCACMGCTATCCTCG	SdhD gene
Dt_SdhD_R	TGGACACTTTAGCTTGTACTTTGCG	SdhD gene
SdhB _HRM_F	CTCAACAACAGCATGAGCTTG	SdhB HRM
SdhB _HRM_R	CAGCGCAGGGTTAAGTCC	SdhB HRM
SdhC_HRM_1F	CCATCTCACCATCTACCGC	SdhC HRM
SdhC_HRM_1R	GTAGAGGGAGCCGGAGAGC	SdhC HRM
SdhC_HRM_79_F	CGTCATTCAACCGTATCACCC	SdhC HRM
SdhC_HRM_2F	CTGAAGAGCTTCTTTGCGTTC	SdhC HRM
SdhC_HRM_135_F1	CCATGTTCTTCCACAGG	SdhC HRM
SdhC_HRM_135_F2	CCATGTTCTTCCACCGC	SdhC HRM
SdhC_HRM_2R	AGCAAATGCCTAACACCATTTGTTG	SdhC HRM
SdhC_HRM_QR_R	CCTAACACCATTGAAGCGTTG	SdhC HRM
SdhD_HRM_F	ATTCCCCTCACAGTTGCC	SdhD HRM
SdhD_HRM_R	TGATGCACGACTCGAAACC	SdhD HRM
SdhD_HRM_112_F	GCTGAACCCCGTCACCGAR	SdhD HRM

The consensus sequences of all isolates for each gene were aligned using MAFFT v7.308 [68] to identify polymorphisms between isolates. The coding sequence of each *D*. *tanaceti Sdh* gene was inferred following alignment of the consensus sequences to *A*. *alternata* strain AR-SBL-4S, *A*. *solani* strain 1178-W1 [27], *C*. *cassiicola* strain IbCor0008 [25] and *Mycosphaerella graminicola* strain R39-1 [16] *Sdh*B, *Sdh*C and *Sdh*D genes (GenBank acc *SdhB*: KC517310, AB548738, JF916687; *Sdh*C: KC517313, AB548741, JF916699; *Sdh*D: KC517315, AB548743, JF916693).

The Sdh genotype of each *D*. *tanaceti* isolate was compared to the boscalid EC_50_ range (μg a.i/mL) assigned by Hay *et al*. [7] from *in vitro* boscalid testing.

### Collection of *Didymella tanaceti* field isolates for fungicide resistance genotyping and population responses

Additional *D*. *tanaceti* isolates were included in this study to further examine *Sdh* genotype variations and validate the predicted *in vitro* fungicide response. Firstly, a random set of *D*. *tanaceti* (*n* = 173) isolates collected from a range of commercial Tasmanian pyrethrum fields since 2004 was obtained for HRM assay development. Secondly, a *D*. *tanaceti* population (*n* = 774) collected by Pearce *et al*. [28] from four pyrethrum fields sown in 2011 within the pyrethrum cropping region across northern Tasmania was used to evaluate the field frequency of each Sdh substitution. Diseased leaves were sampled from each field in August and November 2012. Between the sampling periods, each field received the standard spring fungicide program, which included one application of boscalid (Filan, BASF) [28]. No other SDHI fungicides were applied in this period. Sampling in August was conducted the week prior to the application of the first fungicide in the program. Sampling of diseased material in November occurred 7 to 9 weeks after the final fungicide application of the spring program. Locations sampled within fields were recorded using GPS to allow precise re-sampling in November. Isolation, storage and DNA extraction of *D*. *tanaceti* from disease material was undertaken as outlined by Pearce *et al*. [28]. Mating-type idiomorph and genotype based on simple sequence repeat (SSR) markers were known for each isolate based on previously research [28], allowing for the population dynamics behind the identified Sdh substitutions to be examined.

### HRM assay for *Sdh* allele detection

An HRM assay that detects multiple mutations within the amplified regions [69, 70] and can identify isolates at low frequency and/or novel Sdh substitutions for further *in vitro* boscalid testing, was designed. For assay development, the program uMELT [71] was used for each known polymorphic loci to predict the discrimination of alleles of potential PCR products of varying sizes using an *in silico* HRM algorithm. In instances where the melting temperature was unable to clearly differentiate the known *Sdh* alleles, protocols were adapted to include multiplexed HRM or HRM followed by restriction enzyme digest modifications. Prior to analysing using HRM, the differential product amplification in isolates of each allele, due to the multiplexing primers, was tested using standard PCR. The specific *Sdh* allele of isolates exhibiting novel melt peaks identified during HRM development were sequenced to identify new mutations and HRM assays redesigned as required.

Mutations in the *Sdh*B were detected using a two-step process. This involved HRM analysis followed by restriction digestion and subsequent HRM (Fig 3A) due to a low melt temperature differential between two of the known alleles. The first step involved real-time PCR amplification and HRM with primers SdhB_HRM_F and SdhB_HRM_R (Table 4) that amplified an 85-bp product containing codons 277 and 279 of the *SdhB*. Conditions for amplification were an initial denature of 5 min at 95°C, followed by 40 cycles of denaturation at 95°C for 10 s, annealing at 50°C for 20 s and extension at 72°C for 10 s. Products were melted at 0.2°C intervals from 76 to 90°C with a 10 s hold at each temperature prior to data acquisition. Following HRM, 0.3 U of the restriction enzyme *TaaI (HpyCH4III*, Thermo Scientific, Waltham, MA), 1.5 uL of 10 × Buffer Tango and 3.47 uL of nuclease free water was added to each tube and tubes incubated in the Rotor-Gene for 1 hr at 65°C. *TaaI* recognizes CAN^GT sites. As this site was only found in individuals with a GTT (V) sequence at codon 279, the product from these individuals was digested into two fragments. To identify samples with a positive digest a re-melt protocol consisting of 95°C for 5 minutes and 40°C for 90 s was used to create heteroduplexes of all amplicons prior to melting the products over the same range as previous (Fig 3A). Isolates with a positive digest exhibited a distinct melt (Fig 3A). Analysis of the data of the two melts was undertaken and combined to give the final allele call.

Two independent multiplexed HRM reactions were used to detect concurrent substitutions within the *SdhC* gene (Fig 3B & 3C). For the first reaction, the primers SdhC_HRM_1F and SdhC_HRM_1R (Table 4, Fig 3B) were used to amplify a 88-bp product containing codons 73 and 79. Due to a low temperature differential between isolates with WT and mutation at codon 79, the allele-specific primer SdhC_HRM_79_F (Table 4, Fig 3B) was designed to anneal within the amplified fragment of isolates with the mutation in codon 79, resulting in two products of differing size and thus two melting events in these isolates (Fig 3B). HRM conditions included annealing at 60°C, 35 cycles and melting from 70–90°C in 0.2°C increments. For the second reaction, the primers SdhC_HRM_2F and SdhC_HRM_2R were used to amplify a 62-bp fragment containing codons 134 and 135. To discriminate all the mutations additional primers were designed to anneal within the amplified region. The allele-specific primers SdhC_HRM_135_F1 and SdhC_HRM_135_F2 were included to bind to isolates with a S135R substitution produced from an AGG and CGC mutations, respectively, resulting in two products for these isolates (Table 4, Fig 3C). Additionally, the primer SdhC_HRM_QR_R was designed to amplify a second product in isolates containing the combined H134Q/S135R substitution (Table 4, Fig 3C). HRM conditions included annealing at 55°C, 40 cycles and melting from 68 to 86°C in 0.2°C increments.

To detect mutations in the *SdhD* gene the primers SdhD_HRM_F and SdhD_HRM_R were designed to amplify a 112-bp product containing codons 112 and 122 (Table 4; Fig 3D). Additionally, due to a low temperature differential between alleles, the primer SdhD_HRM_112_F was included to amplify the two mutations resulting in substitution at codon 112 (Table 4. Fig 3D). HRM conditions included annealing at 58°C, 40 cycles and melting from 78 to 91°C in 0.1°C increments.

The HRM assays were conducted using the 2012 *D*. *tanaceti* population to select isolates for *in vitro* boscalid testing and to examine the population structure underlying the substitutions. All HRM reactions were undertaken in a Rotor-Gene Q Real-Time PCR machine (Qiagen). Reaction mixes contained 1 × Type- it HRM buffer (Qiagen), 0.35μM of each primer, 2 ng of DNA and enough nuclease free water to produce a final reaction volume of 10 μL. Within each run, three independent isolates with each known mutation were included as replicate positive controls. Data analysis of each run was undertaken in the Rotor Gene ScreenClust HRM software [72]. For analysis of each run, data was normalised within left and right boundaries of 1°C in width spanning a 10–20°C window over which melting occurred. An “unsupervised” analysis, using *K*-means and the known-genotype isolates (controls) employed as pseudo-unknowns, was used to identify any new putative genotypes, which separated into discrete clusters or appeared as outliers within clusters. The number of clusters and principle components (PC) were determined using the gap statistic [73]. Following the identification of all genotypes, samples were genotyped using the “supervised” mode. The known-genotype isolates were used to calculate a cluster distribution using linear discriminant analysis (LDA), with the centre of each cluster equal to the mean of the known-genotypes isolate of each identified mutation. For each isolate the probability of belonging to each genotype cluster was calculated. Isolates with low probability (<0.1) were selected for repeat HRM and analysis for confirmation. Additionally, 10% of isolates were randomly selected and screened in duplicate to evaluate reproducibility of the HRM assays.

The HRM results were used to select up to ten *D*. *tanaceti* isolates of each Sdh substitution and WT from the population for *in vitro* boscalid testing to confirm the phenotypic response associated with each substitution. Where more than ten isolates of each substitution were identified, SSR profile and mating-type data (based on Pearce *et al*. [28] or unpublished data) were used to select genetically diverse isolates for boscalid testing. Ten WT isolates were included to identify a baseline response of *D*. *tanaceti* isolates. To confirm the substitutions and further validate the HRM assay, the *Sdh*B, *Sdh*C and *Sdh*D of the selected isolates (*n* = 92) were amplified and sequenced. Representative sequences of each mutation were deposited in GenBank (accessions: MK500737—MK500762; Table 1).

### *In vitro* boscalid testing

For *in vitro* testing commercial grade boscalid (Filan; 50% a.i, BASF, Ludwigshafen, Germany) was dissolved in distilled autoclaved water and made up to 75% (*v/v*) in methanol to sterilise and provide an initial stock solution. Dilutions of the stock solution were amended to potato dextrose agar (PDA; Amyl Media, Dandenong, Australia) to obtain final boscalid concentrations of: 0, 0.001, 0.01, 0.05, 0.25, 0.5, 1.25, 2.5, 5, 10, 25 and 50 μg a.i/mL. The final concentration of methanol within each plate did not exceed 0.08% (*v*/*v*). Based on previously obtained EC_50_ range data [7], isolates of each substitution were tested on 6 or 7 boscalid concentrations in the ranges of 0 to 1.25 μg a.i/mL (WT isolates), 0 to 2.5 μg a.i/mL (SdhB-I279V isolates), 0 to 10.0 μg a.i/mL (SdhD-D112E isolates) or 0.0 to 50.0 μg a.i/mL (all remaining isolates with SdhB, SdhC and SdhD substitutions) (Table 1). For testing, a 4 mm diameter plug was excised from the margin of an actively growing 12-day old culture of each *D*. *tanaceti* isolate growing on PDA and placed onto plates of each fungicide concentration in triplicate and incubated at 21°C in the dark. Mycelial growth was measured across two perpendicular lines after 7 d incubation for each replicate plate of each fungicide concentration. Colony diameters were corrected by subtracting the diameter of the agar plug. For each isolate the average colony radius was calculated for each tested concentration. Relative growth at each concentration was calculated as the corrected colony radius divided by the averaged corrected radius of the non-amended controls (0 μg ai/ml). EC_50_ values were calculated using logistic regression of the relative growth against log_10_ of the boscalid concentration in the package NPLR [74] and calculation of the concentration that resulted in reduction of growth by 50% relative to non-amended controls. To allow inclusion of the data from the 0 μg ai/mL, the concentration was transformed, converting it to concentrations 2 log units lower than the lowest tested concentration. Data was analysed in R v3.1.3 [75]. For each isolate Resistance Factors (RFs) were also calculated using the following formula: RF = EC_50_X/EC_50_WT, where EC_50_X is the EC_50_ value of the isolate being examined, and EC_50_WT is the average EC_50_ value of the baseline isolates (WT). A phenotype was inferred based on the interpretation of RF values whereby isolates were grouped as sensitive (RF ≤ 3) or low (3 > RF ≤ 10), moderate (10 > RF ≤ 100), high (100 > RF ≤ 1000) or very high (> 1000) in resistance.

### Frequency and genetic diversity of Sdh substitutions in 2012 *D*. *tanaceti* population

For the *D*. *tanaceti* population sampled in 2012, the frequency (%) of each substitution in each field was calculated for the two sampling periods and overall. Changes in the occurrence of Sdh substitutions between the two sampling periods was assessed by multinomial logistic regression where month of sampling, field of origin and the interaction between these factors were used as fixed effects. To avoid detrimental effects on model predictions, Sdh substitutions that occurred at frequencies less than 1% across the population were excluded from the data set prior to analysis. Analysis was conducted using the nnet package [76] within the R statistical language framework. Post hoc treatment comparisons were conducted with model least-squares means estimates and Tukey’s adjustment for multiple pairwise comparisons, using the package lsmeans [77]. Additionally, the Sdh substitutions were used to define sub-groups within the population. To examine the amount of genetic diversity within each of the sub-groups, the Sdh substitution data was combined with the SSR profile and mating-type data of each isolate identified by Pearce *et al*. [28]. For each sub-group the number of associated multilocus genotypes (MLGs), derived by combining the SSR profile and mating-type data, was calculated for the two sampling periods and overall. The number of genetically unique individuals in the overall population was also calculated by combining the Sdh, SSR and mating-type data.
